# Immunomodulatory effects of curcumin on macrophage polarization in rheumatoid arthritis

**DOI:** 10.3389/fphar.2024.1369337

**Published:** 2024-02-28

**Authors:** Tingting Deng, Jiahe Xu, Qiong Wang, Xing Wang, Yi Jiao, Xiaoxue Cao, Qishun Geng, Mengxiao Zhang, Lu Zhao, Cheng Xiao

**Affiliations:** ^1^ Institute of Clinical Medicine, China-Japan Friendship Hospital, Beijing, China; ^2^ Peking University China-Japan Friendship School of Clinical Medicine, Beijing, China; ^3^ China-Japan Friendship Clinical Medical College, Beijing University of Chinese Medicine, Beijing, China; ^4^ Graduate School of Peking Union Medical College, Chinese Academy of Medical Sciences/Peking Union Medical College, Beijing, China; ^5^ China-Japan Friendship Hospital, Capital Medical University, Beijing, China; ^6^ Department of Emergency, China-Japan Friendship Hospital, Beijing, China

**Keywords:** macrophage polarization, epigenetic regulation, curcumin, rheumatoid arthritis, autoimmune diseases

## Abstract

Rheumatoid arthritis (RA) is a systemic autoimmune disease characterized by synovial inflammation, cartilage destruction, pannus formation and bone erosion. Various immune cells, including macrophages, are involved in RA pathogenesis. The heterogeneity and plasticity of macrophages render them pivotal regulators of both the induction and resolution of the inflammatory response. Predominantly, two different phenotypes of macrophages have been identified: classically activated M1 macrophages exacerbate inflammation via the production of cytokines, chemokines and other inflammatory mediators, while alternatively activated M2 macrophages inhibit inflammation and facilitate tissue repair. An imbalance in the M1/M2 macrophage ratio is critical during the initiation and progression of RA. Macrophage polarization is modulated by various transcription factors, epigenetic elements and metabolic reprogramming. Curcumin, an active component of turmeric, exhibits potent immunomodulatory effects and is administered in the treatment of multiple autoimmune diseases, including RA. The regulation of macrophage polarization and subsequent cytokine production as well as macrophage migration is involved in the mechanisms underlying the therapeutic effect of curcumin on RA. In this review, we summarize the underlying mechanisms by which curcumin modulates macrophage function and polarization in the context of RA to provide evidence for the clinical application of curcumin in RA treatment.

## 1 Introduction

Rheumatoid arthritis (RA) is one of the most common systemic autoimmune diseases affecting approximately 1% of the population worldwide (Venetsanopoulou et al., 2022). RA is characterized by synovitis, pannus formation, cartilage destruction, and progressive bone erosion. Although the etiology of RA has not been fully elucidated, it is thought to be related to complex interactions among the immune system, epigenetic changes, and environmental factors underlying chronic inflammation (Romao and Fonseca, 2021).

As essential components of the innate immune system, macrophages perform multiple functions in inflammation, including the removal of invading pathogens, phagocytosis of apoptotic cells, production of proinflammatory cytokines, and facilitation of antigen presentation for cellular immunity activation. Moreover, during the resolution of inflammation, macrophages play pivotal roles in tissue repair (Fujiwara and Kobayashi, 2005). These functions are due to the plasticity of macrophages (Shapouri-Moghaddam et al., 2018; Yunna et al., 2020). To date, two main classes of macrophages have been identified based on their markers: classically activated M1 macrophages, which play a proinflammatory role, and alternatively activated M2 macrophages, which have anti-inflammatory functions (Murray, 2017). It has become clear that a balance in M1/M2 macrophage polarization is of great significance for the maintenance of immune homeostasis. During the acute phase of inflammation, macrophages are predominantly activated to differentiate into M1 macrophages, which might facilitate the elimination of pathogens through a burst in cytokine production. Following pathogen elimination, macrophage switching leads to an increase in M2 macrophages, which release anti-inflammatory cytokines and growth factors to favor the repression of inflammation and tissue recovery (Wynn et al., 2013). Dynamic changes in macrophage polarization determine the progression and outcome of various inflammatory diseases; therefore, appropriate regulation of macrophage polarization is necessary (Li et al., 2019). An imbalanced M1/M2 macrophage ratio leads to organ damage in multiple autoimmune disorders, including RA, and therapies that contribute to promoting macrophage polarization from the M1 to the M2 phenotype might be new strategies for alleviating inflammation in patients with RA (Cutolo et al., 2022).

Curcumin, the primary active component of turmeric, is a natural compound. It has been extensively demonstrated to possess anti-inflammatory, antioxidant, immunomodulatory and anticancer properties in both experimental and clinical studies (Xu et al., 2018). Curcumin has shown strong therapeutic potential, especially in autoimmune diseases, such as RA and systemic lupus erythematosus (SLE) (Yang et al., 2019; Chamani et al., 2022; Kou et al., 2023). A large number of investigations have indicated that curcumin modulates macrophage polarization and function to alleviate inflammation and therefore can be used to treat inflammation-related diseases (Gao et al., 2015; Karuppagounder et al., 2016; Abdollahi et al., 2023).

In this review, we focus on macrophage polarization and its critical role in the pathogenesis of RA, primarily highlighting the dysregulation of the M1/M2 macrophage balance induced excessive inflammatory responses that are mediated by proinflammatory cytokines and chemokines. Moreover, the therapeutic effect of curcumin on RA is also analysed, particularly the modulatory effect of curcumin on macrophage polarization, which leads to the suppression of proinflammatory cytokine secretion, the switching of M1 macrophages to M2 macrophages, and the inhibition of macrophage migration. There are few comprehensive reviews explaining the regulatory effects of curcumin on macrophage polarization in the context of RA. A thorough understanding of the effect and mechanism of action of curcumin will aid in the development of new strategies for treating RA.

## 2 Functions of macrophage polarization in rheumatoid arthritis

Macrophage polarization refers to the process by which macrophages acquire distinct functional phenotypes in response to a specific microenvironment. Depending on their different activation states, macrophages are involved not only in the progression of inflammation but also in its resolution (Mosser and Edwards, 2008). In RA, the inflammatory process is mediated and sustained by M1 macrophages both in peripheral blood and synovial tissue. In contrast, alternatively activated M2 macrophages contribute to vasculogenesis and tissue remodelling (Cutolo et al., 2022).

### 2.1 M1 macrophage biology

**FIGURE 1:**
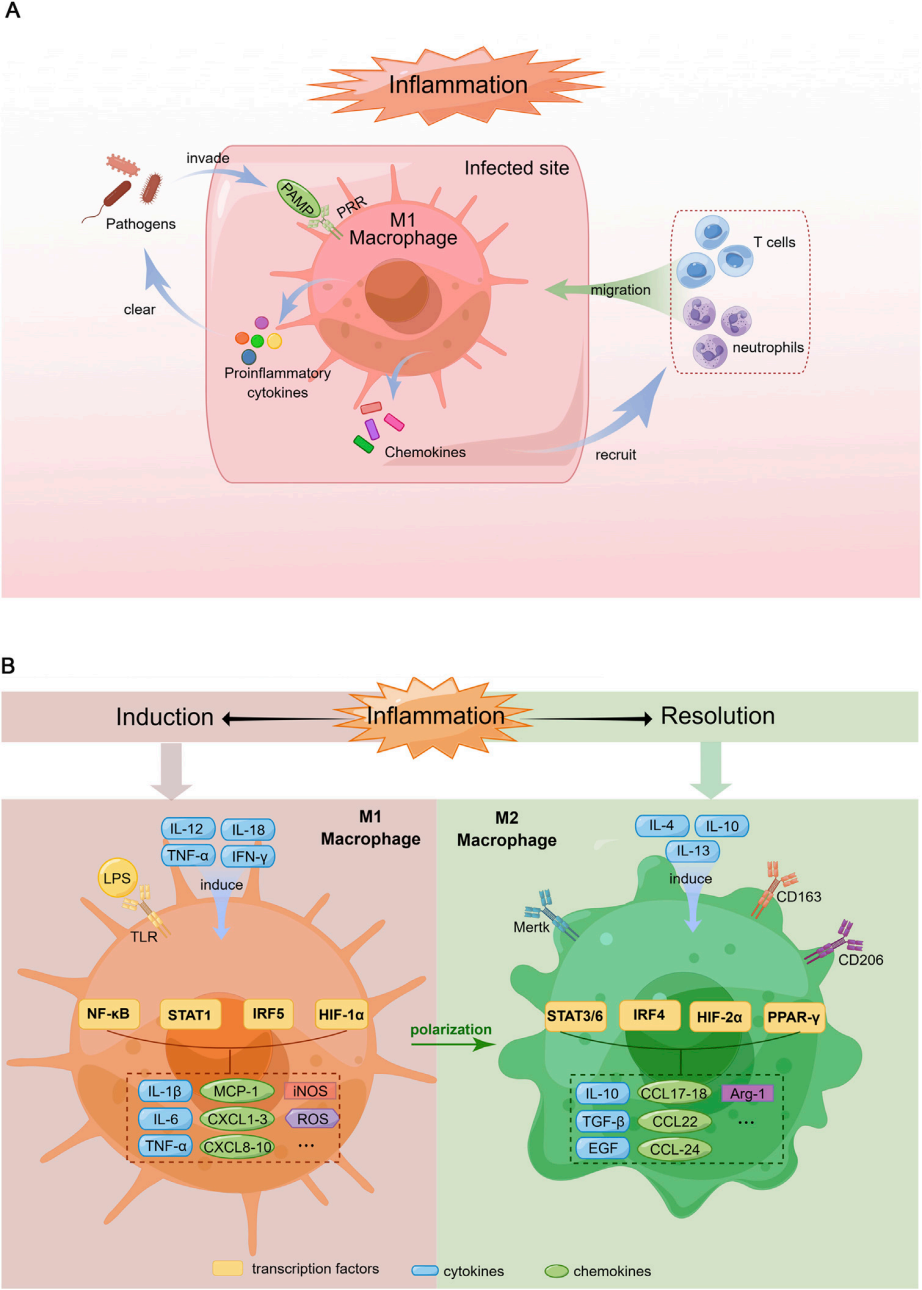
Schematic diagram of macrophage response during the induction and resolution of inflammation. **(A)**. Pro-inflammatory response of macrophages in the context of infection. When pathogens invade the body, PAMPs are recognized by corresponding PRRs, which induce macrophage polarization toward M1 phenotype. Activated M1 macrophages produce proinflammatory cytokines to clear the pathogens and chemokines that recruit more immune cells such as neutrophils and T cells migrating to the infected site, which can amplify inflammatory response thereby remove pathogens rapidly. **(B)**. Polarization of M1 and M2 macrophages. Left: Macrophages are induced to M1 phenotype by the Th1 cytokines (e.g., IL-12, TNF-α, IFN-γ) and LPS. M1 macrophages produce proinflammatory cytokines (IL-1β, IL-6, TNF-α), chemokines (MCP-1, CXCL1-3, 8-10), iNOS and ROS via the activation of multiple transcription factors (NF-κB, STAT1, IRF5, HIF-1α). Right: M2 macrophage differentiation is stimulated by Th2 cytokines (e.g., IL-4, IL-10, IL-13). M2 macrophages secrete anti-inflammatory cytokines (IL-10), growth factors promoting tissue repair (TGF-β, EGF), chemokines (CCL17-18, 22, 24) and Arg-1 through the activation of various transcription factors including STAT3/6, IRF4, HIF-2α and PPAR-γ.

The mechanisms underlying macrophage phenotype modulation and function have not yet been elucidated. Briefly, upon pathogen invasion, macrophages rapidly recognize pathogen-associated molecular patterns (PAMPs) through pattern recognition receptors (PRRs) such as Toll-like receptors (TLRs) and secrete proinflammatory cytokines to clear pathogens while recruiting other immune cells to infected sites by producing chemokines. Therefore, during infection, peripheral blood-derived macrophages are polarized to acquire the M1 phenotype (Figure 1A). However, M1 macrophage activation must be tightly regulated; otherwise, excessive inflammation induced by M1 macrophages could cause tissue destruction, resulting in autoimmune disorders (Ma et al., 2019).

The polarization of macrophages towards the M1 phenotype is typically induced by T helper type 1 (Th1) cell-produced cytokines, such as interleukin (IL)-12, IL-18, tumor necrosis factor (TNF)-α and interferon (IFN)-γ. In addition, PAMPs such as lipopolysaccharide (LPS) are known to promote M1 phenotype differentiation (Yunna et al., 2020) (Figure 1B).

M1 macrophages secrete an array of proinflammatory cytokines, including IL-1, IL-6, IL-12, TNF-α and various chemokines, including CCL-2, which are also known as monocyte chemoattractant protein-1 (MCP-1), CXCL1-3, CXCL5 and CXCL8-10. In addition, these cells produce reactive oxygen species (ROS) and inducible nitric oxide synthase (iNOS) (Shapouri-Moghaddam et al., 2018) (Figure 1B). Hence, M1 macrophages exhibit robust antimicrobial activity and mediate ROS-induced tissue damage.

### 2.2 M2 macrophage biology

Corresponding to the M1 phenotype, M2 macrophages, which were identified in the early 1990s (Stein et al., 1992), are induced by Th2 cell-produced cytokines, such as IL-4, IL-10 and IL-13. Macrophages with this phenotype produce IL-10, transforming growth factor (TGF)-β and epidermal growth factor (EGF), which contribute to inflammation resolution and wound healing (Yunna et al., 2020). The arginase-1 (Arg-1) enzyme, which can degrade L-arginine (a substrate of iNOS), eventually leading to the suppression of T-cell responses, has also been detected in M2 macrophages (Rath et al., 2014). Furthermore, M2 macrophages recruit granulocytes, Th2 cells and regulatory T cells by secreting the chemokines CCL17, CCL18, CCL22 and CCL24 (Porta et al., 2015) (Figure 1B).

The markers expressed on the M2 macrophage surface include scavenger receptors (CD163 and CD204) (Law et al., 1993; Kaku et al., 2014), mannose receptor-1 (CD206) (Porcheray et al., 2005), and Mer tyrosine kinase (Mertk) (Zizzo and Cohen, 2015) (Figure 1B). The main function of CD163 is the removal of haemoglobin-haptoglobin complexes from circulating blood during intravascular haemolysis (Kristiansen et al., 2001). Moreover, CD163 participates in the immunomodulatory process during inflammation, which includes but is not limited to sensing bacteria, binding TNF-like weak inducer of apoptosis (TWEAK) and producing antioxidative substances (Fabriek et al., 2009; Moreno et al., 2009). CD206 is a mannose scavenger receptor that is critical for collagen internalization and degradation (Madsen et al., 2013). Notably, Mertk is essential for the phagocytosis of apoptotic cells by macrophages and functions as a negative regulator of inflammation by binding to its ligand, either growth arrest-specific gene 6 (Gas6) or Protein S (Giroud et al., 2020).

According to their different stimuli and functions, M2 macrophages are further categorized into four subsets: M2a, M2b, M2c and M2d macrophages (Hao et al., 2012). M2a macrophages are activated by IL-13 and IL-4 along with the Th2 cell immune response. M2b macrophages are induced by immune complexes, TLR agonists or IL-1 receptor ligands and play immunomodulatory roles. M2c macrophages are triggered by glucocorticoids and IL-10 and are involved in tissue remodelling. In addition, M2c macrophages express high levels of Mertk, which facilitates their efficient phagocytosis of apoptotic cells. M2d macrophages also known as tumor-associated macrophages (TAMs), are activated by growth factors and exert immunosuppressive effects (Murray and Wynn, 2011; Wang et al., 2019). Each subset has its own biomarkers: CCL17 is a biomarker of M2a macrophages; CCL1 is a biomarker of M2b macrophages; CXCL13 is a biomarker of M2c macrophages (Tsuchimoto et al., 2015); and CD206, Ym1, Fizz1, dectin-1, and arginase-1 are biomarkers of M2d macrophages (Ferrante et al., 2013).

### 2.3 Role of M1/M2 macrophages in RA

#### 2.3.1 Humans

In RA patients, abundant macrophages are widespread in the peripheral blood, synovial fluid and synovial tissue (Roszkowski and Ciechomska, 2021), playing important roles during the initial, active and remission phases of RA.

Blood monocytes are the circulating precursors of macrophages and osteoclasts in RA. Three subsets of monocytes, namely, classical monocytes (CD14^++^CD16^−^), intermediate monocytes (CD14^++^CD16^+^), and non-classical monocytes (CD14^+^CD16^++^), were identified according to CD markers (Ziegler-Heitbrock et al., 2010; Kapellos et al., 2019). Classical monocytes seem to differentiate into osteoclasts and cause bone erosions in RA synovial joints (Komano et al., 2006). The number of intermediate monocytes that are prone to differentiate into M1-macrophages increases in both the peripheral blood and synovia of RA patients. These cells contribute to synovial inflammation by secreting pro-inflammatory cytokines, such as TNF-α, IL-1β and IL-6 (Amoruso et al., 2016). Non-classical monocytes are involved in the early inflammatory response, while later on polarize into resident M2 macrophages and participate in the resolution of inflammation (Thomas et al., 2015).

CD14^+^ monocytes were purified from the peripheral blood of RA patients and healthy controls, and the expression of macrophage polarization markers was evaluated. The results showed that there was no significant difference in the expression of M1 or M2 markers between the two groups, indicating that there was a mixture of M1 and M2 macrophages in the peripheral blood of RA patients (Quero et al., 2017; Zhao et al., 2017).

The synovium is the main site of joint inflammation in RA patients, where inflammatory mediators produced by various cells lead to cartilage destruction, pannus formation and bone erosion. Healthy synovial tissue is composed of a lining-layer enriched with resident macrophages and fibroblast-like synoviocytes (FLSs), alongside a sublining connective tissue containing blood vessels, fibroblasts, adipocytes, and a limited number of resident macrophages (Smith et al., 2003). The macrophages populated in the lining layer predominantly express M2 markers (CD163, CD206 and MerTK) and are involved in the maintenance of synovial homeostasis. Conversely, an increase in macrophages in the sublining layer serves as an early hallmark of synovitis in RA. These cells have heterogeneous phenotypes, with a coexpression of both M1 and M2 markers (Ambarus et al., 2012), thereby indicating a clear distinction between tissue-resident (lining) and infiltrating (sublining) macrophage phenotypes.

Prolonged M1 macrophage activation and an increased M1/M2 macrophage ratio have been found in RA patients. Excessive proinflammatory cytokines and ROS produced by M1 macrophages induce tissue injury during the active phase of RA (Fukui et al., 2017). The activation of TLR4-triggered nuclear factor (NF) -κB signalling mediates the inflammatory response of M1 macrophages by producing IL-1β, IL-6, IL-12 and TNF-α in both monocyte-derived and synovial resident macrophages (Brizzolara et al., 2013). In the early stage of RA, these mediators facilitate the recruitment of monocyte-derived macrophages from the peripheral blood into synovial tissue, thereby perpetuating inflammation. Furthermore, an increase in ROS production occurs in inflamed joints, which exacerbates oxidative tissue damage. The presence of M1 macrophages in RA synovitis can indicate disease activity, so a decrease in M1 macrophages at the target organ level may be a good biomarker of therapeutic response (Tardito et al., 2019).

In contrast to patients with active disease, the synovial tissue of RA patients in remission is characterized by a greater presence of M2 macrophages. Some of the metabolic pathways activated in M2 macrophages contribute to the anti-inflammatory effect of these cells in RA. IL-10, IL-12 and TGF-β1 secreted by M2 macrophages inhibit the production of proinflammatory factors and limit inflammation. In addition, promoting the transformation of macrophages from the M1 to the M2 phenotype in RA helps to inhibit the formation of osteoclasts and weaken the erosion of articular cartilage and bone, and consequently improve the condition of patients, which may shed light on discovering new drugs for treating RA (Chandran and Goel, 2012; Tsuchimoto et al., 2015; Cutolo et al., 2022).

#### 2.3.2 Mice

Studies on murine models of RA illustrate mouse counterparts of human monocytes, which include Ly6C^++^CD43^+^ monocytes (equivalent to human classical monocytes), Ly6C^++^CD43^++^ monocytes (equivalent to intermediate monocytes) and Ly6C^−^ monocytes (equivalent to non-classical monocytes) (Ingersoll et al., 2010). Both Ly6C^++^CD43^+^ and Ly6C^++^CD43^++^ monocytes promote sterile joint inflammation. Similar as in humans, non-classical monocytes (Ly6C^−^) initially differentiate into M1 macrophages contributing to the progression of joint inflammation and later on, polarized into M2 macrophages, promoting inflammation resolution (Misharin et al., 2014).

Macrophages in the lining layer that selectively express CX3CR1 form a protective epithelial-like barrier that is responsible for the maintenance of tissue hemeostasis. Using K/BxN serum-transfer arthritis (STA) and collagen-induced arthritis (CIA) mouse models, rapid changes in morphology and spatial orientation without the proliferation of CX3CR1^+^ macrophages were observed at the onset of inflammation. In contrast, the number of CX3CR1^−^ interstitial macrophages, which also express MHCII, increases rapidly, and these cells may actively contribute to joint inflammation (Culemann et al., 2019).

### 2.4 Mechanisms underlying macrophage polarization

#### 2.4.1 Transcriptional modulation

**FIGURE 2:**
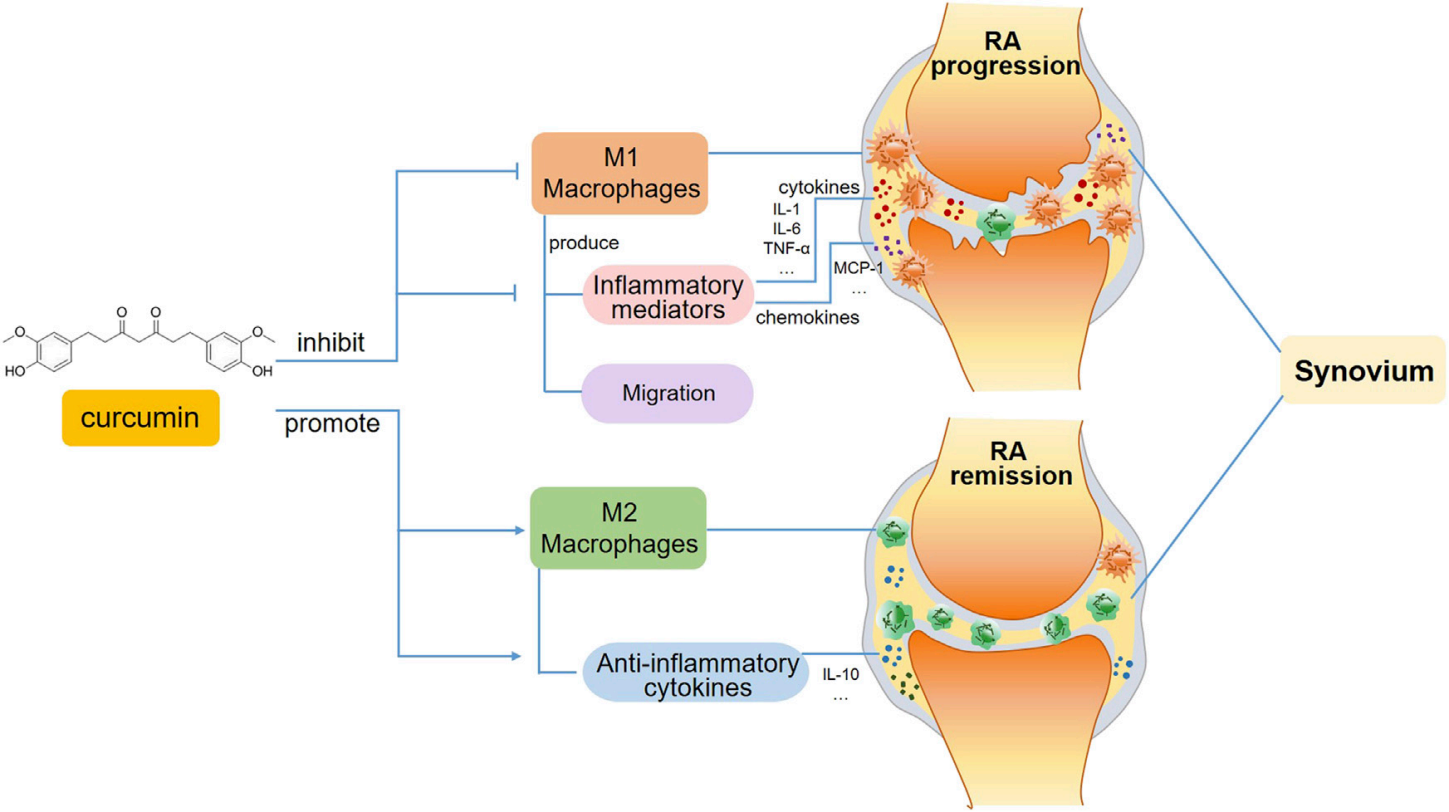
A schematic view of curcumin’s immunomodulatory effects on macrophages in RA. In the progression phase of RA, curcumin inhibits M1 macrophage polarization and subsequent production of inflammatory mediators, including pro-inflammatory cytokines and chemokines. Moreover, the migration of M1 macrophages is also suppressed. Whereas in the remission phase of RA, curcumin favors inflammation resolution and tissue recovery via promoting M2 macrophage polarization.

The signalling pathways related to macrophage polarization have not yet been fully elucidated. Nevertheless, transcription factors such as NF-κB, the STAT family, IFN regulatory factors (IRFs), hypoxia-inducible factors (HIFs) and peroxisome proliferator-activated receptor (PPAR)-γ have been demonstrated to be key regulatory molecules of this process (Xue et al., 2014). Specifically, the M1 phenotype is regulated by STAT1, IRF5 and HIF-1α, whereas STAT3, STAT6, IRF4, HIF-2α and PPARγ are involved in M2 macrophage polarization (Lawrence and Natoli, 2011) (Figure 2).

Multiple signalling pathways including the NF-κB (Simmonds and Foxwell, 2008), mitogen-activated protein kinases (MAPKs), Notch (Keewan and Naser, 2020), and phosphatidylinositol-3-kinase (PI3K)/Akt (Qi et al., 2019) pathways, have been reported to participate in the regulation of macrophage polarization in RA. NF-κB is a pivotal transcription factor that participates in inflammatory response of macrophages, and activation of NF-κB signalling strongly promotes M1 macrophage polarization (Lin et al., 2020). It was confirmed that punicalagin inhibited M1 phenotype polarization through the suppression of NF-κB signalling, which eventually alleviated joint inflammation, cartilage damage and systemic bone destruction in CIA mice (Ge et al., 2022). Similar results were obtained in another study using Lonicerin as a therapeutic compound. *In vivo* and *in vitro* studies revealed that Lonicerin significantly decreased M1 marker levels by attenuating the activation of the NF-κB signalling pathway, which contributes to M1 macrophage polarization and inflammasome activation (Yang et al., 2023).

Another important pathway involved in M1 macrophage polarization is MAPKs signalling, which includes the ERK1/2, JNK, and p38 kinases. Downregulation of the phosphorylation levels of ERK, JNK, and p38 by nintedanib inhibited M1 macrophage polarization in the inflamed synovium (Yan et al., 2023).

Notch signalling, which is associated with multiple cellular processes, including survival, proliferation, differentiation and metabolism, has been implicated to favor M1 macrophage polarization, which leads to the overexpression of TNF-α, IL-6, and MCP-1. On the other hand, emerging evidence indicates the role of Notch signalling in the pathogenesis of RA (Nakazawa et al., 2001; Keewan and Naser, 2020). A study using (TNF-)-transgenic/(Hes-1)-GFP mice identified M1 macrophages derived from bone marrow as the main cells with active Notch signalling in the inflamed joints of RA mice, while thapsigargin, a Notch inhibitor, reduced TNF-induced M1 macrophage polarization and alleviated joint lesions by switching M1 macrophages to M2 macrophages (Sun et al., 2017).

Increasing evidence suggests that the PI3K/Akt pathway also plays pivotal roles in macrophage polarization. PI3K regulates a host of cellular functions, including cell viability, metabolism, motility, and proliferation through the activation of downstream kinases. Akt, which is composed of three members (Akt1, Akt2, and Akt3), is the most prominent effector of PI3K (Ersahin et al., 2015). Several compounds inhibiting PI3K/Akt signalling have therapeutic effects on the progression of RA. Cassiaside C naphthopyrone prevents LPS/IFN-γ-induced M1 macrophage polarization through the inhibition of PI3K/AKT/mTORC1 signalling (Kim et al., 2022). Hesperidin inhibits synovial cell inflammation and macrophage polarization through suppression of the PI3K/AKT pathway in an adjuvant-induced arthritis (AIA) mouse model (Qi et al., 2019).

#### 2.4.2 Epigenetic modulation

In addition to transcriptional regulation, increasing evidence has shown that epigenetic modifications, including DNA methylation, RNA methylation, noncoding RNA actions, and histone modification, play important roles in the modulation of macrophage polarization. DNA methyltransferase (DNMT) 1 is related to proinflammatory gene expression and M1 macrophage activation. Another DNMT, DNMT3b, is also involved in the polarization of M1 macrophages, which has been confirmed by result demonstrating that the knockdown of DNMT3b leads to the switch of M1 macrophages into M2 macrophages (Yang et al., 2014). Therefore, DNMT inhibitors (DNMTis) such as 5-azacytidine can be utilized for inflammatory disease treatment. In recent years, RNA epigenetic modifications have been highlighted as a novel class of epigenetic regulatory events. N6-methyladenosine (m6A) is the most abundant epigenetic modification of mammalian mRNAs. Notably, the m6A-catalytic enzyme methyltransferase like 3 (METTL3) promotes M1 polarization of mouse macrophages by directly methylating STAT1 mRNA (Liu et al., 2019). METTL3 is also involved in bone repair by targeting histone deacetylase 5 (HADC5) to affect macrophage polarization (Lei et al., 2021). It has been demonstrated that depletion of the m6A demethylase fat mass and obesity-associated protein (FTO) inhibits the NF-κB signalling pathway and reduces the mRNA stability of PPAR-γ and STAT1 via YTHDF2-mediated degradation, thereby significantly suppressing the polarization of M1 and M2 macrophages simultaneously (Gu et al., 2020). The m6A reader insulin-like growth factor 2 messenger RNA (mRNA)-binding protein 2 (IGF2BP2) can bind to TSC1 and PPARγ directly to skew M1 macrophages towards M2 activation via the TSC1-mTORC1 pathway and PPARγ mediated fatty acid uptake (Liu et al., 2021). A recent study indicated that the m1A “reader” YTHDF3 may participate in modulating macrophage polarization, which promotes aortic inflammation and influences abdominal aortic aneurysm progression by regulating the expression of its target genes (Wu et al., 2022). Emerging evidence has shown that miRNAs modulate macrophage polarization and subsequently affect inflammation (Liu and Abraham, 2013). MiR-9, miR-127, miR-155 and miR-125b have been shown to promote M1 polarization, while miR-124, miR-223, miR-34a, let-7c, miR-132, miR-146a and miR-125a-5p induce M2 polarization in macrophages by targeting various transcription factors (Essandoh et al., 2016). Histone acetylation occurs when macrophages are stimulated by TLR activation, after which the expression of multiple proinflammatory cytokine genes is upregulated. Similarly, trimethylation of histone 3 lysine 4 on cytokine gene promoters has also been shown to be induced in M1 macrophages via TLR stimulation, suggesting that histone modification is triggered during the process of M1 macrophage activation leading to inflammatory gene expression (Takeuch and Akira, 2011).

#### 2.4.3 Metabolic modulation

The metabolism of macrophages is closely related to their phenotype. From a metabolic perspective, M1 macrophages rely mainly on glycolysis, which can meet the requirements of biosynthetic intermediates in the inflammatory response. In contrast, M2 macrophages are more dependent on oxidative phosphorylation, which supports anti-inflammatory processes (Viola et al., 2019). It has been demonstrated that Cassiaside C can dampen M1 polarization of macrophages by downregulating glycolysis (Kim et al., 2022). The existing evidence suggests that macrophages of RA patients have a distinctive metabolic signature that favors aerobic glycolytic metabolism (Jutley et al., 2021). Therefore, metabolic reprogramming through the inhibition of glycolysis in M1 macrophage may be an effective strategy to switch the polarization to the M2 phenotype in RA treatment (Freemerman et al., 2014). Berberine, an isoquinoline alkaloid isolated from Chinese herbs, was found to inhibit M1 macrophage polarization to ameliorate joint inflammation in CIA mice through the activation of AMP-activated protein kinase to switch glycolytic reprogramming (Cheng et al., 2023).

## 3 Therapeutic effect of curcumin on RA

### 3.1 Biological activities of curcumin

Curcumin is a natural compound isolated from the rhizome of the plant *Curcuma longa* (turmeric) that has been used to treat inflammation, cancer and neurodegenerative diseases such as multiple sclerosis (MS) and Parkinson’s disease for centuries. Intensive studies carried out within the past 3 decades confirmed that the anti-inflammatory and antitumour properties of turmeric are attributable to its active component, curcumin (Arora et al., 1971). A large number of studies including both animal model experiments and clinical trials, have verified the anti-inflammatory and immunomodulatory properties of curcumin (Ferguson et al., 2021; Peng et al., 2021; Chamani et al., 2022). Curcumin regulates the functions of various immune cells, including macrophages (Mohammadi et al., 2019), dendritic cells (DCs) (Rahimi et al., 2021), B cells (Mohammadi et al., 2022) and T cells (Rahimi et al., 2019), thereby modulating both innate and adaptive immunity (Shehzad and Lee, 2013). The anti-inflammatory activity of curcumin is due to its suppression of multiple signalling molecules, including NF-κB, activated protein (AP)-1, MAPKs, and protein kinase C (Kahkhaie et al., 2019). In addition, curcumin is a potent inhibitor of reactive-oxygen-generating enzymes, such as lipoxygenase, cyclooxygenase (COX), xanthine dehydrogenase and iNOS (Rao, 2007). As shown in macrophages, curcumin inhibits LPS and IFN-γ-induced nitric oxide production (Brouet and Ohshima, 1995).

Curcumin has been demonstrated to be safe even when it is administered at high doses. A phase 1 human trial in which as much as 8000 mg of curcumin per day was administered for 3 months to patients with high-risk or premalignant lesions reported no toxic effects (Cheng et al., 2001). No serious side effects were reported in RA patients receiving 500 mg of curcumin per day over a period of 8 weeks (Chandran and Goel, 2012). Curcumin treatment has no obvious toxic effect on liver or kidney functions; therefore, curcumin is generally recognized as a safe compound by the U.S. Food and Drug Administration (Xu et al., 2018).

However, there is a problem in the application of curcumin; that is, it shows poor aqueous solubility and chemical instability, leading to low bioavailability (Anand et al., 2007). Several strategies have been developed to enhance curcumin bioavailability, including delivery via nano/microparticles, lipid-based nanocarriers, adjuvants with piperine, solid dispersions, etc (Ma et al., 2019). The efficacy of curcumin delivery via nanotechnology has been assessed in preclinical and clinical trials established to evaluate treatments for RA (da Silva et al., 2019; Javadi et al., 2019).

### 3.2 Curcumin in the treatment of RA

The immune system has evolved to react only to foreign antigens while maintaining tolerance to self-antigens (Goldrath and Hedrick, 2005). However, under certain conditions, dysregulation of the immune system, which induces prolonged chronic inflammation, results in the development of inflammatory diseases, including RA. Abnormally activated immune cells such as Th1 cells and M1 macrophages produce large quantities of cytokines, chemokines, ROS, and other inflammatory molecules; moreover, apoptotic cells cannot be eliminated in a timely manner, ultimately resulting in tissue damage (O'Shea et al., 2002). Therefore, the treatment strategy for RA mainly involves suppressing the inflammatory response and promoting of tissue repair. Recent studies have described the effectiveness of using neutralizing antibodies against cytokines or drugs that modulate the polarization of macrophages to control the progression of RA (Hurlimann et al., 2002; Fischer et al., 2015; Yang et al., 2020).

#### 3.2.1 Cultured cells

Numerous *in vitro* studies have addressed the potent inhibitory effect of curcumin on the inflammatory response of synovial fibroblasts (Pourhabibi-Zarandi et al., 2021) and immune cells (Mohammadian Haftcheshmeh et al., 2021). Curcumin has been confirmed to inhibit PGE2 production, COX-2 expression, and matrix metalloproteinases (MMPs) secretion by suppressing NF-κB transcriptional activity in FLSs (Moon et al., 2010). Pre-treatment of RA FLSs with curcumin before stimulation with TNF-α significantly reduced the expression of IL-6, IL-8, MMP-1, and MMP-3 at the protein level (Ahn et al., 2015). A study using RAW264.7 cells indicated that bisdemethoxycurcumin could inhibit LPS-induced proinflammatory cytokine production and cell migration (Sun et al., 2024). Another *in vitro* study reported that curcumin treatment suppressed the differentiation of naive CD4^+^ T cells into Th1 cells through the inhibition of IL-12 (Kang et al., 1999) and IL-18 (Yadav et al., 2015) produced by macrophages.

#### 3.2.2 Animal models

An increasing number of studies have confirmed the effectiveness of curcumin in treating animal models of RA, in which uncontrolled inflammation is a major player. A study showed that curcumin attenuated the degree of joint swelling and further promoted the development of joint histopathology in CIA rats, which are models of RA (Wang et al., 2019). Another study suggested that curcumin treatment alleviated the main symptoms associated with the pathogenesis of CIA, such as inflammation and synovial hyperplasia. The underlying mechanisms may involve the inhibition of mTOR signalling by curcumin and the subsequent production of proinflammatory cytokines, including IL-1β, TNF-α, MMP-1, and MMP-3 (Dai et al., 2018).

#### 3.2.3 Humans

In recent years, an increasing number of randomized clinical trials have been conducted to assess the efficacy and safety of curcumin treatment in patients with RA. Treatment with 500 mg of curcumin twice daily for 8 weeks dramatically improved the Disease Activity Score 28 (DAS-28) and American College of Rheumatology (ACR) score (ACR-20, 50 and 70), which were significantly better than those of patients in the diclofenac sodium group (Chandran and Goel, 2012). A novel curcumin formulation with increased bioavailability significantly improved the DAS-28 score, erythrocyte sedimentation rate (ESR), C-reactive protein (CRP) level, visual analogue scale (VAS) score, rheumatoid factor (RF) level, and ACR response at a dose as low as 250 mg (Amalraj et al., 2017). Similar results were acquired when using another novel hydrogenated curcuminoid formulation, CuroWhiteTM, once daily for 3 months (Jacob et al., 2019). A randomized controlled trial which investigated the effect of curcumin supplementation on metabolic parameters, inflammatory factors, visfatin levels, and obesity values in women with RA, revealed that the homeostatic model assessment for insulin resistance, the ESR, serum levels of CRP and triglycerides, weight, body mass index, and the circumference of patients decreased significantly in the curcumin group, indicating a modulatory effect of curcumin on metabolism and inflammation (Pourhabibi-Zarandi et al., 2022). More importantly, no significant side effects were observed in the patients in these studies. Accumulating systematic reviews have thoroughly analysed clinical trials that were carried out in recent years to evaluate the efficacy and safety of curcumin for the treatment of RA (Zeng et al., 2022b; a; Kou et al., 2023; Long et al., 2023). A meta-analysis of 10 clinical trials covering 539 RA patients indicated that curcumin supplementation improved the ESR, CRP, DAS, RF, VAS, tender joint count and swollen joint count in these patients. Similar results were obtained in another systematic review, in which curcumin was found to significantly improve morning stiffness, walking time, and joint swelling in RA patients. Several mechanisms, including the inhibition of MAPK, ERK1/2, AP-1, and NF-κB have also been reported (Pourhabibi-Zarandi et al., 2021). Overall, the results suggest that curcumin is an effective and safe drug for RA therapy.

### 3.3 Modulation of macrophage function and polarization by curcumin in RA

The alleviation of RA by curcumin might be associated with the inhibition of proinflammatory cytokines produced by M1 macrophages, which is mediated by multiple transcription factors, such as NF-κB, AP-1 and the STAT family (Abdollahi et al., 2018).It has been reported that curcumin regulates inflammatory reactions by inhibiting COX-2 activity, which suppresses the secretion of the proinflammatory cytokines TNF-a, IL-1, and IL-6 (Kunnumakkara et al., 2017) (Figure 2).

Moreover, curcumin has been found to induce the polarization of macrophages, which switch from the M1 to the M2 phenotype, thereby alleviating the progression of RA. A study using an AIA model indicated that the administration of curcumin either alone or in combination with methotrexate apparently alleviated paw inflammation in rats, and this mechanism might be related to the suppression of M1 macrophage polarization and the promotion of M2 macrophage differentiation (Abd-Elhalem et al., 2023) (Figure 2). An *in vitro* study revealed that curcumin inhibited M1 macrophage polarization via the JAK-STAT pathway, thereby further reducing inflammation-mediated apoptosis of osteocytes (Jin et al., 2020). The modulatory effect of curcumin on macrophage polarization has also been demonstrated in other autoimmune diseases. For example, curcumin inhibited LPS-induced neuroinflammation by promoting the differentiation of microglia, which are macrophages that reside in the central nervous system (CNS), and play pivotal roles in CNS autoimmune diseases such as MS and neuromyelitis optica, towards the M2 phenotype, and this effect was attributed to the suppression of the TREM2/TLR4/NF-κB pathways by curcumin (Zhang et al., 2019). Curcumin ameliorated experimental autoimmune myocarditis by upregulating the expression of classical M2 markers, including the macrophage mannose receptor, Arg-1 and PPAR-γ. The induction of M2 macrophage polarization was mediated by increasing IL-4 and IL-13 expression and promoting STAT6 phosphorylation (Gao et al., 2015).

In addition to its effect on macrophage polarization, curcumin appears to regulate macrophage migration (Figure 2). When pathogens invade the body, monocytes/macrophages need to travel to the infected site timely, where they facilitate the rapid removal of pathogens via direct phagocytosis and proinflammatory cytokine secretion. The inhibition of macrophage migration could also serve as a potential anti-inflammatory strategy for treating RA. The migration of monocytes/macrophages is mediated by various chemokines, such as MCP-1 and macrophage inflammatory protein-1β. As a key mediator of inflammatory processes, MCP-1 modulates the migration and tissue infiltration of circulating monocytes/macrophages through its receptor CCR2. In this regard, MCP-1 may be a potential target in anti-inflammatory therapies (Melgarejo et al., 2009). The nanoemulsion curcumin significantly reduced macrophage recruitment via the inhibition of NF-κB p65 subunit phosphorylation and MCP-1 expression (Young et al., 2014). Bisdemethoxycurcumin, a curcumin derivative, has been shown to reduce LPS-stimulated migration in macrophages (Sun et al., 2024).

## 4 Conclusion and perspectives

As a natural compound, curcumin is favorable for improving symptoms in RA patients due to its potent efficacy, affordability, and minimal side effects. The therapeutic potential of curcumin in halting RA progression has been confirmed, which might be associated with the inhibition of cytokines induced by M1 macrophages, the promotion of macrophage polarization towards the M2 phenotype, and the suppression of macrophage migration. Despite these promising findings, several challenges remain with regard to clinical application, such as determining the effective dosage for different phases of RA and enhancing bioavailability to improve therapeutic outcomes. Therefore, further randomized clinical trials are imperative to establish the optimal dosages and administration methods of curcumin for RA treatment in the future.

Although the therapeutic effects of curcumin on RA development have been demonstrated, the underlying mechanisms have not been fully elucidated and are worthy of further investigation. Previous studies have focused mainly on the regulation of transcription factors such as NF-κB and STATs, and the inhibition of downstream inflammatory cytokine production (Bright, 2007; Chamani et al., 2022). The polarization of macrophages plays significant roles in the initiation, progression and remission of RA. However, relatively few studies have focused on the mechanism underlying the ability of curcumin to modulate the polarization of macrophages, especially through the promotion of M2 macrophage differentiation, which probably functions as an upstream regulator and plays a fundamental role during the process. Future studies should delve into this aspect to better understand the mechanism of action of curcumin before it is applied in the treatment of RA patients.

As mentioned above, epigenetic regulation plays crucial roles in macrophage polarization, highlighting epigenetic modifications as noteworthy targets in the treatment of RA. Our previous study established a transcriptional map of m6A in peripheral blood mononuclear cells (PBMCs) from RA patients, demonstrating a significant correlation between changes in RNA methylation and RA-related genes (Fan et al., 2022). Furthermore, our latest study investigated the role and regulatory mechanisms of hypoxia-induced expression of the m6A demethylase alkB homologue 5 (ALKBH5) in RA FLSs, revealing that the HIF1α/2α-ALKBH5-CH25H axis may be crucial for FLS aggression and inflammation (Fan et al., 2024). These findings underscore the link between epigenetic modulation and RA pathogenesis. In addition, curcumin has been reported to ameliorate several diseases via epigenetic regulation, encompassing: (1) suppression of DNMTs; (2) regulation of histone acetyltransferases and histone deacetylases; and (3) regulation of miRNAs (Boyanapalli and Kong, 2015). Several studies have shown that curcumin can be used in cancer treatment to reverse DNA methylation, alter histone modifications and target miRNA expression (Shu et al., 2011; Bao et al., 2012; Yu et al., 2013). Nevertheless, relatively few studies have been conducted to explore the therapeutic mechanism underlying the effects of curcumin in RA from the perspective of epigenetic regulation, which is a novel and promising field that deserves more attention.

## Figures and Tables

**Table:** 

**ACR**	American College of Rheumatology
**AIA**	Adjuvant-induced arthritis
**ALKBH5**	AlkB homologue 5
**AP**	Activated protein
**Arg-1**	Arginase-1
**CIA**	Collagen-induced arthritis
**CNS**	Central nervous system
**COX**	Cyclooxygenase
**CRP**	C-reactive protein
**DAS-28**	Disease Activity Score 28
**DNMT**	DNA methyltransferase
**EGF**	Epidermal growth factor
**ESR**	Erythrocyte sedimentation rate
**FLSs**	Fibroblast-like synoviocytes
**FTO**	Fat mass and obesity-associated protein
**Gas6**	Growth arrest-specific gene 6
**HADC5**	Histone deacetylase 5
**HIFs**	Hypoxia-inducible factors
**IFN**	Interferon
**IGF2BP2**	Insulin-like growth factor 2 messenger RNA -binding protein 2
**IL**	Interleukin
**iNOS**	Inducible nitric oxide synthase
**IRFs**	IFN regulatory factors
**LPS**	Lipopolysaccharide
**m6A**	N6-methyladenosine
**MAPKs**	Mitogen-activated protein kinases
**MCP-1**	Monocyte chemoattractant protein-1
**Mertk**	Mer tyrosine kinase
**METTL3**	Methyltransferase like 3
**MMP**	Matrix metalloproteinases
**MS**	Multiple sclerosis
**PAMPs**	Pathogen-associated molecular patterns
**PBMCs**	Peripheral blood mononuclear cells
**PI3K**	Phosphatidylinositol-3-kinase
**PPAR**	Peroxisome proliferator-activated receptor
**PRRs**	Pattern recognition receptors
**RA**	Rheumatoid arthritis
**RF**	Rheumatoid factor
**ROS**	Reactive oxygen species
**SLE**	Systemic lupus erythematosus
**STA**	Serum-transfer arthritis
**TAMs**	Tumor-associated macrophages
**TGF**	transforming growth factor
**Th1**	T helper type 1
**Th2**	T helper type 2
**TNF**	Tumor necrosis factor
**TLRs**	Toll-like receptors
**TWEAK**	TNF-like weak inducer of apoptosis
**VAS**	Visual analogue scale
